# The effect of topical airway anesthesia on hemodynamic profiles during the induction period in patients undergoing cardiac surgery: Study protocol for a randomized controlled trial

**DOI:** 10.3389/fcvm.2022.992534

**Published:** 2022-10-10

**Authors:** Wenya Du, Meng Lv, Tingting Chen, Xiaxuan Sun, Jihua Wang, Haixia Zhang, Chuansong Wei, Yi Liu, Changlong Qiao, Yuelan Wang

**Affiliations:** ^1^Department of Anesthesiology, The First Affiliated Hospital of Shandong First Medical University & Shandong Provincial Qianfoshan Hospital, Shandong Institute of Anesthesia and Respiratory Critical Care Medicine, Jinan, China; ^2^Shandong First Medical University & Shandong Academy of Medical Sciences, Jinan, China

**Keywords:** topical airway anesthesia, atomization inhalation, anesthesia induction, cardiac surgery, hemodynamics

## Abstract

**Background:**

Patients scheduled for cardiac surgery are often accompanied by cardiac dysfunction and hemodynamic instability. However, the conventional induction strategy for anesthesia using high-dose intravenous anesthetics is often associated with persistent and recurrent hypotension after tracheal intubation. The purpose of this study is to investigate the effects of topical airway anesthesia on the hemodynamic profile of patients undergoing cardiac surgery during the induction period.

**Methods:**

This is a superiority, single-blind, randomized controlled study with two parallel groups. Participants scheduled to undergo elective cardiac surgery will be allocated into two blocks according to the New York Heart Association (NYHA) classification and then randomly assigned to the following two groups at a 1:1 ratio: the conventional induction group and the combined topical airway anesthesia induction group. The combined topical airway anesthesia induction strategy includes aerosol inhalation airway anesthesia, subglottic airway anesthesia, and general anesthesia induction using low-dose intravenous anesthetics. The primary outcome is the area under the curve (AUC) of blood pressure below baseline mean arterial pressure (MAP) from 3 to 15 min after endotracheal intubation. Secondary outcomes include the AUC above baseline MAP and below baseline MAP at other time points, the highest and lowest arterial blood pressure values during the induction period, type and dose of vasoactive drugs, incidence of arrhythmias, cardiac function, and the incidence of postoperative hoarseness and sore throat.

**Discussion:**

The study will explore whether aerosol inhalation airway anesthesia and subglottic airway anesthesia could reduce the incidence and duration of hypotension during the induction period in patients undergoing cardiac surgery.

**Clinical Trial Registration:**

This trial was registered on www.ClinicalTrials.gov (NCT05323786).

## Introduction

Anesthesia induction is one of the most challenging periods in cardiac surgery, especially in patients with left ventricular dysfunction (1). Anesthesia induction is often associated with hemodynamic instability (2), especially recurrent hypotension after tracheal intubation, which could be associated with poor postoperative outcomes (3).

To our knowledge, the ideal anesthesia induction strategy for cardiac surgery patients involves not only to provide adequate depth of anesthesia during endotracheal intubation, but also to stabilize hemodynamics after tracheal intubation. Conventionally, cardiac surgery patients are induced by using high-dose intravenous anesthetics to inhibit endotracheal intubation stress (4). However, this is often accompanied by persistent and recurrent hypotension after endotracheal intubation (5), which requires a large dose of vasoactive drugs to maintain hemodynamic stability. However, high doses of vasoactive drugs are associated with a poor prognosis in patients (6) and can cause serious cardiovascular adverse events (7). Therefore, it is essential to optimize anesthesia induction strategy for patients undergoing cardiac surgery.

Previous studies (8, 9) have shown that atomization inhalation of 2% lidocaine is effective for airway surface anesthesia. It is not clear whether topical airway anesthesia combined with intravenous anesthesia can maintain hemodynamic stability after intubation in patients undergoing cardiac surgery. Thus, such a trial optimizing anesthesia induction strategy using atomization inhalation of 2% lidocaine is warranted.

## Methods and analysis

### Study design

This is a randomized, controlled, single-blind, superiority study, parallel group with 1:1 allocation ratio. This study was approved by the Research Ethics Committee of the First Affiliated Hospital of Shandong First Medical University and was registered on Clinicaltrials.gov (registration number: NCT05323786). This study will be conducted at The First Affiliated Hospital of Shandong First Medical University, Ji'nan City, Shandong Province. All procedures will be performed in accordance with the most recent version of the Declaration of Helsinki.

**Figure 1:**
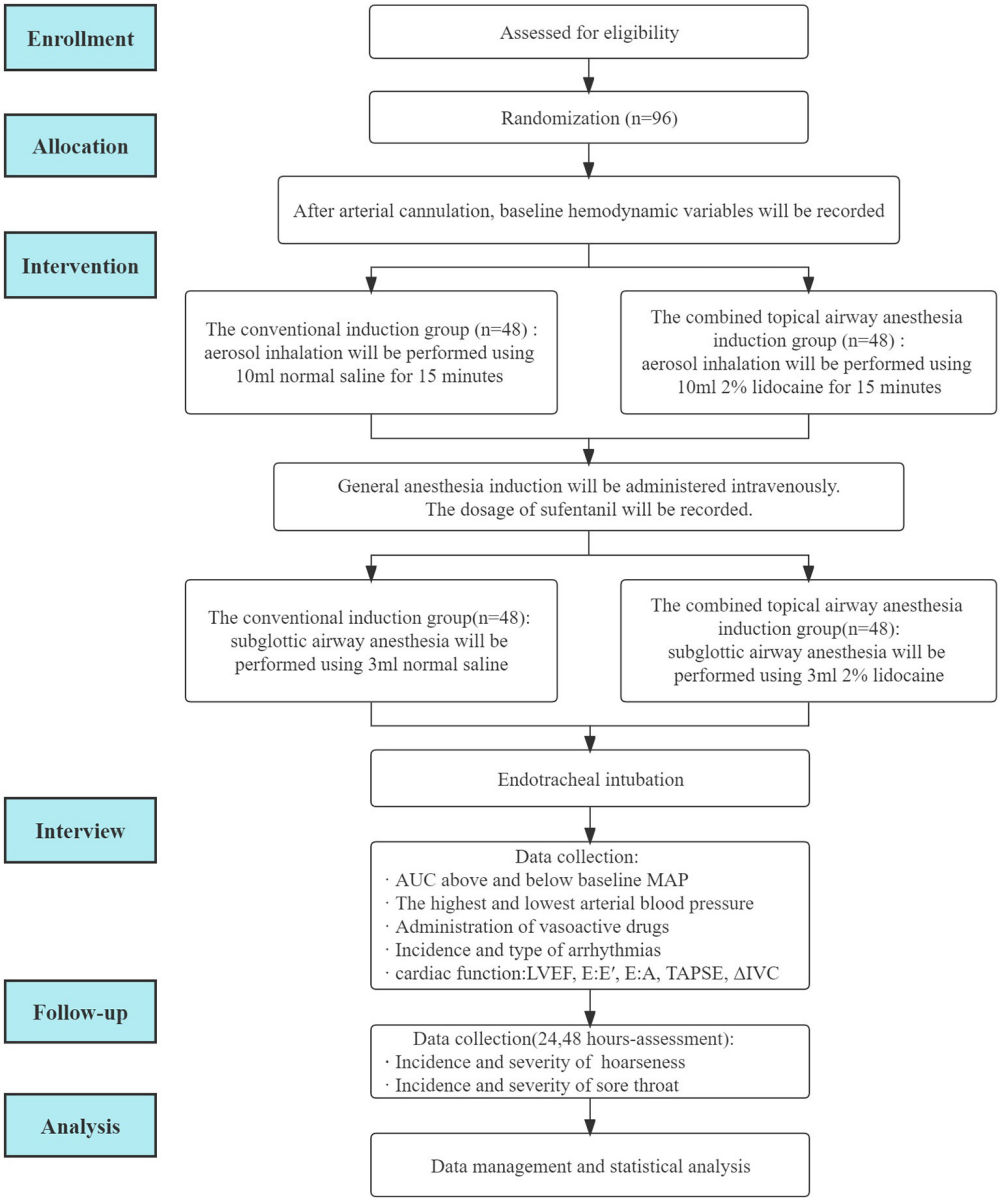
Flowchart of the clinical trial design.

This study protocol will be performed according to the following standard protocol items: Recommendations for Interventional Trials (SPIRIT) statement (10). A flowchart of the clinical trial design is illustrated in Figure 1.

### Participants

From 1 April 2022 to 31 December 2022, participants scheduled to undergo elective cardiac surgery will be recruited from The First Affiliated Hospital of Shandong First Medical University. Participants will be selected for study enrollment according to the inclusion and exclusion criteria prior to surgery. Participants who meet the inclusion criteria and provide signed informed consent will be enrolled in this study. The participant can withdraw from the study at any time (Figure 1).

### Inclusion criteria

The following patients will be enrolled in the study: (1) patients older than 18 years and younger than 75 years; (2) patients scheduled to undergo elective cardiac surgery; (3) patients with New York Heart Association (NYHA) class II–III; and (4) patients providing signed informed consent for the clinical study.

### Exclusion criteria

The following patients will be excluded from the study: (1) patients who unable to cooperate in accepting topical airway anesthesia (with mental disorders or inability to communicate); (2) patients with left heart assist devices prior to surgery; (3) patients with extracorporeal membrane oxygenation (ECMO) prior to surgery; (4) patients with aortic dissection; (5) patients with difficult airway; (6) patients allergic to lidocaine; (7) patients with atrioventricular block; and (8) patients who have participated in other clinical studies during the last 3 months.

### Sample size calculation

A one-sided two-sample *t*-test with a sample size of 39 patients in each group will achieve 80% power to detect a ratio of 0.700 when the ratio under the null hypothesis is 1.000. The coefficient of variation of the original scale is 0.6. Based on Hannam et al. (11), we identified a coefficient of variation of 60% for the AUC mean arterial pressure (MAP) of topical airway anesthesia as clinically significant. We defined a clinically relevant effect as a difference between the groups in AUC (reduction of baseline MAP values of more than 15 min after induction) of ≥33.3%. For this effect size, we estimate that 39 patients per arm would be needed to demonstrate superiority (a < 0.025, one- sided) with 80% power. The potential dropout rate for various reasons is assumed to be 20% and 48 patients in each group were estimated for inclusion, resulting in a total of 96 patients.

### Randomization

**Figure 2:**
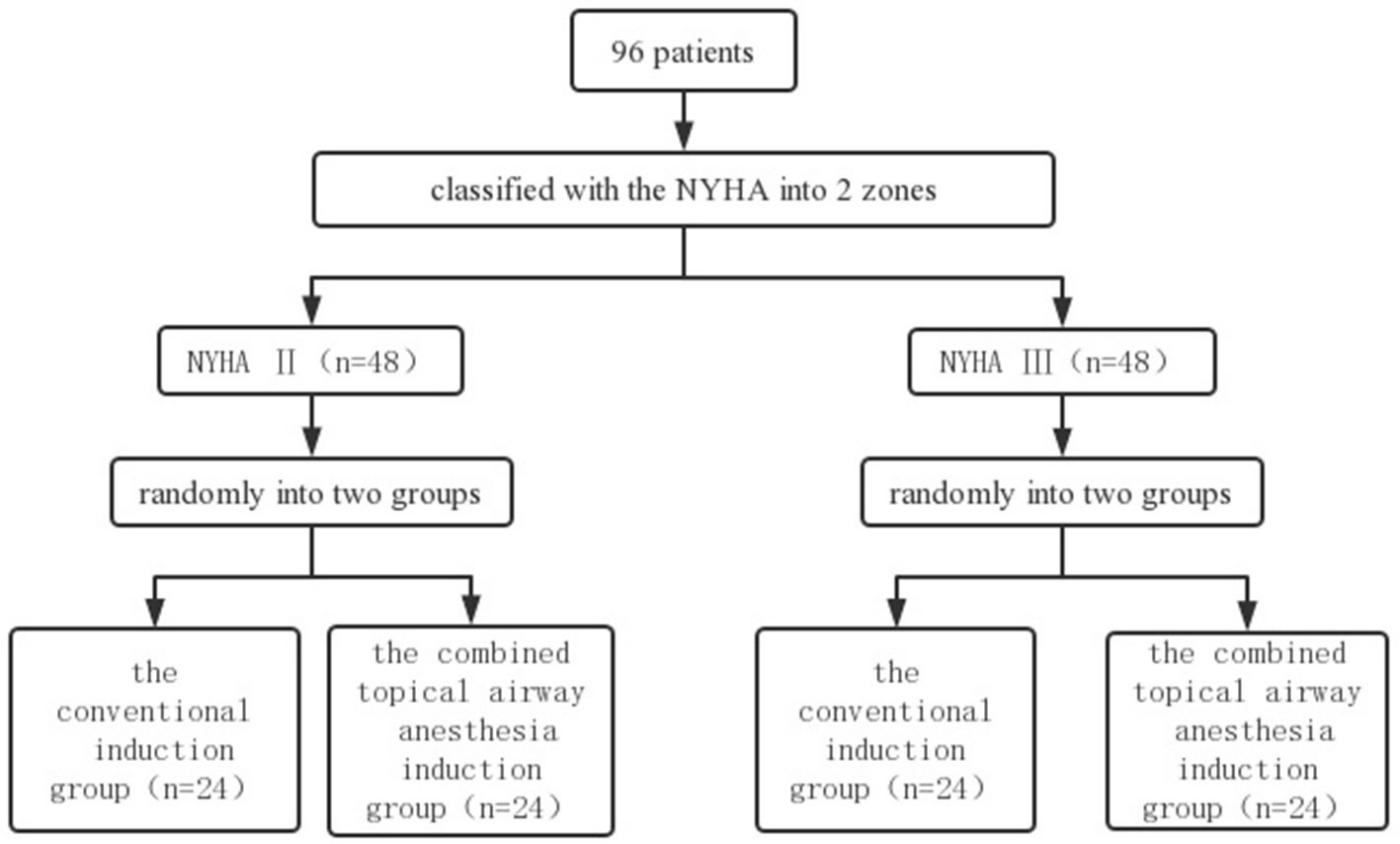
A stratified randomization flow chart.

Participants who provide signed informed consent will be randomly assigned to two blocks according to the New York Heart Association (NYHA) class II–III and will then be randomly assigned to the conventional induction group and the combined topical airway anesthesia induction group within each block in a 1:1 ratio based on a computer-generated random number table (12). The random number table will be generated by an independent investigator using Excel software. A stratified randomization flow chart is shown in Figure 2.

### Allocation concealment

Sequentially numbered, opaque sealed envelopes (SNOSE), locked in the investigator's office, will be used to ensure concealment until participant identification. Participants will be automatically included in the trial when the numbered envelopes are opened, and the numbers on each envelope would ensure that all participants are enrolled after being randomly assigned. Recruitment records will be established, and exclusion of patients will also be recorded.

### Blinding

In this study, a single blind method will be used. The anesthesiologist responsible for the management of perioperative anesthesia will prepare the experimental drugs before the participant enters the room. Participants will be unaware of the group allocation. In addition, researchers responsible for postoperative follow-up and outcome data collection will also be unaware of group allocation. Unblinding will be performed at the end of the statistical analysis. Additionally, emergency blinding will be performed in the event of severe adverse events.

### Intervention

For the combined topical airway anesthesia induction group, 10 mL of 2% lidocaine in a nebulizer and 3 mL of 2% lidocaine in a syringe will be prepared. For the conventional induction group, sham lidocaine (10 mL normal saline) in a nebulizer and 3 mL normal saline in a syringe will be prepared.

After admission to the operating room, participants will be connected to the electrocardiogram (ECG), pulse oxygen saturation, noninvasive blood pressure, and bispectral index (BIS) of the electroencephalograph (EEG). A catheter will be inserted into the radial artery to monitor invasive blood pressure.

Before general anesthesia induction, the combined topical airway anesthesia induction group will receive aerosol inhalation using 10 mL of 2% lidocaine, and the conventional induction group will receive aerosol inhalation using 10 mL of normal saline. Aerosol inhalation will last for 15 min with high flow oxygen (8–10 L/min) (13). The aerosol inhalation will be performed in the 45–60° semi-sitting position (14).

After completion of atomization inhalation, general anesthesia induction will begin. At the beginning of general anesthesia induction, midazolam (0.05 mg/kg) and etomidate (0.2–0.3 mg/kg) will be administered intravenously. When BIS is <50, the anesthesiologist will administer atracurium (0.6 mg/kg) and sufentanil (0.15–0.3 μg/kg). All participants will be in Trendelenburg position (15–30°) during the induction period. In the conventional induction group, the anesthesiologist who is in charge of the intraoperative anesthesia will decide the dosage of sufentanil according to the physical condition of the participant and the hemodynamic changes during the induction period. The dosage of sufentanil will be recorded.

After 3–5 min of administration of intravenous induction drugs, subglottic airway anesthesia will be performed using 3 mL 2% lidocaine (the combined topical airway anesthesia group) or 3 mL normal saline (the conventional induction group) with a tracheal topical anesthesia kit (Tuoren; Henan Province, China).

Tracheal intubation will be performed 3–5 min after subglottic anesthesia. After endotracheal intubation, the anesthesiologist will decide whether to start propofol infusion according to the BIS value and will adjust the infusion rate to maintain the BIS value between 40 and 60. Target-controlled infusion (TCI) with propofol (1 μg/mL) will be initiated.

The hemodynamic variables of the participants, which include arterial blood pressure (systolic blood pressure (SBP), diastolic blood pressure (DBP), MAP and HR, will be recorded at baseline (T0; 3 min after radial arterial cannulation and before the beginning of aerosol inhalation), at the beginning of aerosol inhalation (T1), at the beginning of general anesthesia induction (T2), at the completion of subglottic airway anesthesia (T3), at the completion of tracheal intubation (T4), and during central venous catheterization (T5) (Table 1).

**Table 1:** Time schedule of enrolment, interventions, assessments, and visits for the participants.

**Timepoint**	**Enrolment**	**Allocation**	**Post-allocaton**						**Follow-up**	
	**1**	**2**	**T0**	**T1**	**T2**	**T3**	**T4**	**T5**	**4**	**5**
**Enrolment**										
Eligibility screen	X									
Informed consent	X									
Allocation		X								
**Interventions**										
Conventional induction group					X					
Combined topical airway Anesthesia induction group				X	X	X				
**Assessments**										
1. Area under the curve of blood pressure below baseline			X	X	X	X	X	X		
2. Type,frequency and dosage of vasoactive drugs				X	X	X	X	X		
3. Incidence of arrhythmias				X	X	X	X	X		
4. Cardiac systolic/diastolic function:LVEF,E:E′,E:A,TAPSE			X					X		
5. Respiratory variation in inferior vena cava diameter: ΔIVC			X					X		
6. Number of patients with postoperative hoarseness (GRBAS score)									X	X
7. Number of patients with postoperative sore throat (NRS 0–10)									X	X

Variables related to cardiac systolic and diastolic function and effective blood volume, including left ventricular ejection fraction (LVEF), E:E' (the ratio of the E peak and E'), E:A (the ratio of the E peak and A peak), tricuspid annular plane systolic excursion (TAPSE), and respiratory variation in inferior vena cava diameter (deltaIVC), will be evaluated using a transthoracic probe before induction of the anesthesia and transesophageal echocardiography (TEE) after placement of transesophageal echocardiography (TEE) probe.

### Rescue treatment

During the induction period, the anesthesiologist will decide whether to use vasoactive drugs as needed. After repeated use of the same vasoactive drug more than three times, the vasoactive drug will be continuously infused. The type, times, and total dose of the vasoactive drugs will be recorded. If the MAP or HR increases by more than 15% from baseline and the BIS is >60, the anesthesiologist will administer 30 mg of propofol, which may be administered repeatedly as needed. If the MAP or HR increases by more than 15% from baseline and the BIS is <60, the anesthesiologist will administer 10 mg of urapidil or 10 mg of esmolol, which could be administered repeatedly. If the MAP decreases over 15% from baseline and HR is <50 beats per minute (bpm), 1 mg of dopamine will be administered. If the MAP decreases more than 15% from baseline and HR is >50 bpm, 1 μg norepinephrine will be administered.

### Outcome measures

The outcomes will be evaluated by independent researchers who have been trained prior to the study.

### Primary outcome measure

The primary outcome is the area under the curve (AUC) below baseline MAP from 3 to 15 min after endotracheal intubation, define as the MAP-time integral (11). We define baseline MAP as the average of three times 3 min after radial arterial cannulation with a 1-min interval.

### Secondary outcome measures

Secondary outcomes include the following variables: (1) AUC above baseline MAP (MAP-time integral) and below baseline MAP (MAP-time integral) from the beginning of general anesthesia induction to 3 minutes after endotracheal intubation; (2) the highest and lowest arterial blood pressure (SBP, DBP, MAP) from the beginning of general anesthesia induction to 15 min after endotracheal intubation; (3) administration of vasoactive drugs from the beginning of general anesthesia induction to 15 min after endotracheal intubation, including times, dosage, and type; (4) incidence and type of arrhythmias, including atrioventricular block, atrial fibrillation, and premature beat; (5) preoperative cardiac systolic function, evaluated by LVEF. LVEF% = (left ventricular end diastolic diameter-left ventricular end systolic diameter)/left ventricular end diastolic diameter × 100%; (6) preoperative and intraoperative cardiac diastolic function, represented by E:E' and E:A; (7) TAPSE is a commonly used variable of right ventricular systolic function; (8) effective blood volume, measured by deltaIVC; (9) the incidence and severity of postoperative hoarseness 24 and 48 h after surgery will be evaluated using the Grade, Roughness, Breathiness, Asthenia, and Strain (GRBAS) scale. This tool includes 5 items. Each item is a 4-point scale, ranging from 0, normal to 3, severely impaired (15); (10) the incidence and severity of sore throat 24 and 48 h after surgery. The severity of sore throat will be assessed using a numeric rating scale (NRS): (0–10 score, 0: no pain, 10: worst imaginable pain) (16, 17).

### Data collection

Demographic data, including age, sex, height, and weight, will be recorded. The American Society of Anesthesiologists (ASA) score and the New York Heart Association (NYHA) classification will be evaluated. Patients diagnosed with pre-existing cardiovascular disease (CVD), diabetes mellitus, liver disease, kidney disease, lung disease, or brain disease will be identified. Smoking history, alcohol consumption, and medication history (for example, ACE inhibitors, ARB, or beta-blockers) will also be recorded. Laboratory tests (for example, routine blood test, liver function test, renal function test, coagulation test), electrocardiogram, echocardiographic diagnosis, and coronary angiographic diagnosis will be collected. After admission to the operating room, baseline MAP will be collected. Heart rate, blood pressure, and MAP values will be recorded every 5 s during the post-induction study period. Furthermore, variables related to cardiac systolic and diastolic function (for example, LVEF, E:E', E:A, TAPSE) and effective blood volume (for example, deltaIVC) will be collected before induction and after tracheal intubation.

### Data management

The relevant data from the participants will be collected by independent researchers. Standardized data collection files will be used to ensure completeness and quality. Outcomes will be measured by two researchers who have been trained using a standardized Case Report Form (CRF). All personal data will be stored on a different locked computer in a secure room at the hospital. An Excel database will be established on the basis of the CRF files. Two researchers will complete the relevant information in time and accurately. Data will be transmitted directly by hand from research members to the locked computer, never via mail or the Internet. Once sensitive data are entered into the computer, any written documents that would allow participants to be identified will be destroyed to avoid information leakage. Only researchers will have access to the electronic data.

### Data analysis

All statistical data analyses will be performed using SPSS v25.0 (IBM, Chicago, IL, USA) statistical software. We will perform intention-to-treat and per-protocol analyses for the primary outcome. Normally distributed variables will be expressed as the mean ± standard deviation (SD) and compared with Student's t-test. Categorical variables will be compared using the χ2 test or Fisher's exact test, as appropriate. Non-normal distributed continuous variables will be expressed as median (IQR) and evaluated using the Mann-Whitney U-test. A value of *P* < 0.05 will be considered statistically significant and all reported *P*-values will be two-sided. During the study design period, we consulted the Center for Big Data Research in Health and Medicine with study design, sample size calculation, and statistical analysis plan.

### Handling of loss to follow-up cases

We will make great efforts to limit loss to follow-up within 5%, such as excluding patients who may be unwilling to participate in the follow-up before randomization, completing each follow-up as fast as possible, and streamlining the experimental procedure during follow-up. If the rate of loss to follow-up exceeds 5%, multiple imputations will be performed to deal with incomplete data.

## Discussion

In this single-center, superiority, single-blind, randomized, controlled trial, our aim is to investigate whether topical airway anesthesia combined with intravenous anesthesia can reduce hemodynamic fluctuations during the induction period in patients undergoing cardiac surgery.

Our study has multiple strengths. First, Wieczorek et al. (18) argued that topical airway anesthesia could provide excellent supraglottic and subglottic anesthesia and greatly reduce endotracheal intubation stimulation. Second, our previous studies (12) showed that supraglottic airway anesthesia using a vapouriser could cause nausea and cough. Additionally, the efficacy of supraglottic airway anesthesia is influenced by the degree of cooperation. Topical airway anesthesia with aerosol inhalation of 2% lidocaine using an atomizing mask is not only effective (19), but also more tolerable (20). Third, the study is a well-designed, randomized, and controlled single-blind trial, so internal validity is guaranteed. Our trial has two limitations. First, this study is a single blind trial, which might lead to inadvertent bias. Second, this is a single center study; thus, external validation is limited.

## Ethics statement

The study was reviewed and approved by the Research Ethics Committee of the First Affiliated Hospital of Shandong First Medical University [YXLL-KY-2021(080)]. The participants will provide written informed consent to participate in the trial. The results of this study will be distributed in international peer-reviewed journals.

## Trial status

At the time of protocol manuscript submission, the recruitment of the participants has started and is ongoing.

## Author contributions

ML conceived and designed the study and designed the method for statistical analyses. WD, TC, XS, and CQ will recruit and screen the participants. HZ and CW will participate in performing random assignment concealment and blinding. ML, WD, TC, XS, and CQ will participate in the data collection. ML, WD, JW, and YL will participate in the data analysis. WD and ML participated in drafting this manuscript. YW and ML provided the supervision support. All authors contributed to the article and approved the submitted version.

## Funding

This study was supported by Qilu Special Clinical Research Project from Shandong Medical Association (YXH2022ZX02088).

## Conflict of interest

The authors declare that the research was conducted in the absence of any commercial or financial relationships that could be construed as a potential conflict of interest.

## Publisher's note

All claims expressed in this article are solely those of the authors and do not necessarily represent those of their affiliated organizations, or those of the publisher, the editors and the reviewers. Any product that may be evaluated in this article, or claim that may be made by its manufacturer, is not guaranteed or endorsed by the publisher.

## Abbreviations

NYHA: New York Heart Association
AUC: the area under the curve
MAP: mean arterial pressure
IABP: intra-aortic balloon pump
ECMO: Extracorporeal Membrane Oxygenation
SAP: systolic arterial pressure
BIS: the bispectral index
LVEF: Left ventricular ejection fraction
E:E': the ratio of E peak and E'
E:A: the ratio of E peak and A peak
TAPSE: Tricuspid annular plane systolic excursion
deltaIVC: Respiratory variation in inferior vena cava diameter
GRBAS, grade, roughness, breathiness: asthenia and strain
NRS: numeric rating scale.
