# Corrigendum: The effect of topical airway anesthesia on hemodynamic profile during the induction period in patients undergoing cardiac surgery: Study protocol for a randomized controlled trial

**DOI:** 10.3389/fcvm.2023.1190323

**Published:** 2023-03-31

**Authors:** Wenya Du, Meng Lv, Tingting Chen, Xiaxuan Sun, Jihua Wang, Haixia Zhang, Chuansong Wei, Yi Liu, Changlong Qiao, Yuelan Wang

**Affiliations:** ^1^ Department of Anesthesiology, The First Affiliated Hospital of Shandong First Medical University & Shandong Provincial Qianfoshan Hospital, Shandong Institute of Anesthesia and Respiratory Critical Care Medicine, Jinan, China; ^2^ Shandong First Medical University & Shandong Academy of Medical Sciences, Jinan, China

**Keywords:** topical airway anesthesia, atomization inhalation, anesthesia induction, cardiac surgery, hemodynamics

A Corrigendum on The effect of topical airway anesthesia on hemodynamic profile during the induction period in patients undergoing cardiac surgery: Study protocol for a randomized controlled trial By Du W, Lv M, Chen T, et al. (2022) Front Cardiovasc Med. 9:992534. doi: 10.3389/fcvm.2022.992534

**Incorrect Affiliation**

In the published article, there was an error in affiliation 1. Instead of “Shandong First Medical University, Taian, China” it should be “Department of Anesthesiology, The First Affiliated Hospital of Shandong First Medical University & Shandong Provincial Qianfoshan Hospital, Shandong Institute of Anesthesia and Respiratory Critical Care Medicine, Jinan, China”.

In the published article, there was an error in affiliation 2. Instead of “Department of Anesthesiology and Perioperative Medicine, The First Affiliated Hospital of Shandong First Medical University, Jinan, China” it should be “Shandong First Medical University & Shandong Academy of Medical Sciences, Jinan, China”.

In the published article, there was an error regarding the affiliations for Wenya Du, Meng Lv, Tingting Chen, Xiaxuan Sun, and Yuelan Wang. As well as having affiliation 1^“^Department of Anesthesiology, The First Affiliated Hospital of Shandong First Medical University & Shandong Provincial Qianfoshan Hospital, Shandong Institute of Anesthesia and Respiratory Critical Care Medicine, Jinan, China” they should also have affiliation 2^“^Shandong First Medical University & Shandong Academy of Medical Sciences, Jinan, China”.

In the published article, there was an error regarding the affiliation for Jihua Wang, Haixia Zhang, Chuansong Wei, Yi Liu, and Changlong Qiao. Their affiliation was displayed as “Department of Anesthesiology and Perioperative Medicine, The First Affiliated Hospital of Shandong First Medical University, Jinan, China”. The correct affiliation is “Department of Anesthesiology, The First Affiliated Hospital of Shandong First Medical University & Shandong Provincial Qianfoshan Hospital, Shandong Institute of Anesthesia and Respiratory Critical Care Medicine, Jinan, China”.

The authors apologize for these errors and state that this does not change the scientific conclusions of the article in any way. The original article has been updated.

